# Supplementary material for: Effects of home-based telerehabilitation on dynamic alterations in regional intrinsic neural activity and degree centrality in stroke patients
Source: PeerJ. 2023 Sep 1;11:e15903. doi: 10.7717/peerj.15903 (PMC10476610; doi:10.7717/peerj.15903)
Supplement: Supplemental Information 1 [file peerj-11-15903-s001.docx]

The inclusion criteria were as follows: (1) aged from 30 ~ 85 years; (2) right-handedness before stroke; (3) screening within 1 ~ 3 weeks after stroke symptom onset and patients in a stable condition; (4) first-onset stroke with a single subcortical lesion involving the motor pathway; (5) clinical evidence of hemiplegia based on neurological examination, and the corresponding responsible lesions were evident on CT or MRI; (6) a National Institutes of Health Stroke Scale (NIHSS) score from 2 ~ 20; and (7) patients not receiving regular rehabilitation training yet but who have a strong demand for rehabilitation and good family support.

The exclusion criteria included the following: (1) unconsciousness, cognitive impairment, or cooperation difficulties; (2) cerebellar or pontine lesions; (3) other brain abnormalities or psychiatric disorders, or clinically significant or unstable medical diseases; (4) use of medications that might affect motor examinations, such as antipsychotics and antiepileptics; (5) contraindications for MRI scanning; and (6) patients suffering from claustrophobia.
